# Evaluation of accuracy for detection and extent of occult myocardial scars using delayed-enhancement CT in patients with asymptomatic diabetes: results from the ACCREDIT study

**DOI:** 10.1186/1532-429X-16-S1-P87

**Published:** 2014-01-16

**Authors:** Joon-Won Kang, Sang Il Choi, Sung Min Ko, Joon Ho Choi, Yoonyoung Choi, Jong Chun Park, So Youn Shin, Tae-Hwan Lim

**Affiliations:** 1 Department of Radiology, Asan Medical Center, Seoul, Korea, Republic of; 2 Department of Radiology, Seoul National University Bundang Hospital, Seongnam, Gyeong-gi, Korea, Republic of; 3 Department of Radiology, Konkuk University Hospital, Seongnam, Gyeong-gi, Korea, Republic of

## Background

Occult myocardial scar is known to be a predictive factor of poor prognosis in the diabetes patients. The purposes of this study are to evaluate through an exploratory sub-study the accuracy of delayed-enhancement CT (DE-CT) for detecting occult myocardial scars (OMS) and to evaluate the transmurality of OMS using DE-CT compared with delayed enhancement MRI (DE-MRI) in asymptomatic patients with type 2 diabetes.

## Methods

In this prospective, multicenter, and open-label study, 347 patients with type 2 DM were included with 2 or more risk factors of coronary artery disease. DE-MRI and DE-CT were respectively performed with Gadoterate Meglumine (Dotarem^®^) and Iobitridol (Xenetix^®^350) on 167 patients. Image quality of DE-CT was evaluated using 4-grading system, good, adequate, poor and null. The prevalence and the transmurality of OMS on both DE-MRI and DE-CT at patient level and segment level were evaluated. The sensitivity, specificity, positive and negative predictive values of DE-CT for detecting OMS was evaluated in comparison with DE-MRI both at patient and segment level. The transmurality of OMS in DE-CT and DE-MRI for each scar was assessed in terms of over- or underestimation of DE-CT using six grade system, none, 1-25%, 26-50%, 51-75%, and 76-99%, and 100% at segment level.

## Results

The image quality of DE-CT was good and adequate in 165 (98.8%) patients. The OMS was detected in 12 (7.3%) by DE-MRI and 7 (4.3%) by DE-CT of the 164 patients. Of the 2788 segments, OMS was detected in 24 segments on DE-MRI and in 12 segments on DE-CT. The sensitivity of DE-CT for detecting OMS was 58.3% (7 of 12), specificity was 100% (152 of 152), positive predictive value was 100% (7 of 7) and the negative predictive value was 96.8% (152 of 157) at patient level. And, at segment level, the sensitivity, specificity, positive and negative predictive value of DE-CT for detecting OMS were 50% (12 of 24), 100% (2764 of 2764), 100%, and 99.6% respectively. The grade for transmurality of OMS between DE-CT and DE-MRI was matching at segment level in 41.7% (10 of 24), under-evalutaion of transmurality on DE-CT was in 54.2% (13 of 24), and overestimation on DE-CT was 4.2% (1 of 24).

## Conclusions

The sensitivity of DE-CT for detecting OMS is moderate, but the specificity is high. Under-evaluation of The transmurality of OMS is common using DE-CT.

## Funding

This study is supported by the Guerbet group, France.

**Table 1:** Agreement table between DE-CT and DE-MRI

DE-MRI/ DE-CT	none	1-25%	25-50%	50-75%	75-99%	100%	OMS(+), no grading
none	2764	5	6				1

1-25%		5	1				

25-50%			3				

50-75%			1	1			

75-99%					0		

100%						0	

OMS(+), no grading							1

